# Supplementary material for: Permissive parenting of the dog associates with dog overweight in a survey among 2,303 Dutch dog owners
Source: PLoS One. 2020 Aug 11;15(8):e0237429. doi: 10.1371/journal.pone.0237429 (PMC7418960; doi:10.1371/journal.pone.0237429)
Supplement: S1 Table — Spearman rank correlations were calculated between the original 32-PSDQ parenting styles of authoritative, authoritarian and permissive parenting and the DD-PSDQ parenting styles determined in the previous study by Van Herwijnen et al., 2018 of authoritative-training orientated, authoritative-intrinsic value orientated and authoritarian-correction orientated parenting (N = 2,303, P<0.001 for all). (PDF) [file pone.0237429.s001.pdf]

S1 Table - Spearman rank correlations between the parenting styles directed at the dog

Spearman rank correlations were calculated between the original 32-PSDQ parenting styles of authoritative, authoritarian and permissive parenting and the DD-PSDQ parenting styles determined in the previous study by Van Herwijnen et al., 2018 of authoritative-training orientated, authoritative-intrinsic value orientated and authoritarian-correction orientated parenting (*N*=2,303, P<0.001 for all).

	**Authoritative style**	**Authoritarian style**	**Permissive style**
**Authoritative style**	-	$r_s=-0.27$	$r_s=0.18$
**Authoritarian style**	$r_s=-0.27$	-	$r_s=0.12$
**Permissive style**	$r_s=0.18$	$r_s=0.12$	-

	**Authoritative-training orientated style**	**Authoritative-intrinsic value orientated style**	**Authoritarian-correction orientated style**
**Authoritative-training orientated style**	-	$r_s=0.27$	$r_s=-0.27$
**Authoritative-intrinsic value orientated style**	$r_s=0.27$	-	$r_s=-0.24$
**Authoritarian-correction orientated style**	$r_s=-0.27$	$r_s=-0.24$	-

	**Authoritative-training orientated style**	**Authoritative-intrinsic value orientated style**	**Authoritarian-correction orientated style**
**Authoritative style**	$r_s=0.58$	$r_s=0.85$	$r_s=-0.25$
**Authoritarian style**	$r_s=-0.24$	$r_s=-0.29$	$r_s=0.93$
**Permissive style**	$r_s=-0.17$	$r_s=0.30$	$r_s=0.13$
