# Supplementary material for: Permissive parenting of the dog associates with dog overweight in a survey among 2,303 Dutch dog owners
Source: PLoS One. 2020 Aug 11;15(8):e0237429. doi: 10.1371/journal.pone.0237429 (PMC7418960; doi:10.1371/journal.pone.0237429)
Supplement: S2 Table — A dog’s body condition score (BCS) being underweight (grouping body condition scores one to three), healthy-weight (score four and five) or overweight/obese (score six to nine) was calculated to fall into an owner’s first to fourth quartile of parenting style scores for each of the three parenting styles of authoritative, authoritarian and permissive parenting and the additionally analysed specific styles of authoritative-training orientated, authoritative-intrinsic value and authoritarian-correction orientated parenting. Chi-square tests for these frequencies were significant only for permissive parenting (χ2 = 33.8, P<0.001, df = 6, N = 2,303; all other P>0.001). (PDF) [file pone.0237429.s002.pdf]

S2 Table - Counts of underweight, healthy-weight, overweight/obese dog body condition scores per quartile of an owner's parenting style scores

A dog's body condition score (BCS) being underweight (grouping body condition scores one to three), healthy-weight (score four and five) or overweight/obese (score six to nine) was calculated to fall into an owner's first to fourth quartile of parenting style scores for each of the three parenting styles of authoritative, authoritarian and permissive parenting and the additionally analysed specific styles of authoritative-training orientated, authoritative-intrinsic value and authoritarian-correction orientated parenting. Chi-square tests for these frequencies were significant only for permissive parenting (χ^2=33.8, P<0.001, df=6, *N*=2,303; all other P>0.001).

	**Underweight** *(BCS 1-3)*	**Healthy-weight** *(BCS 4-5)*	**Overweight** *(BCS 6-9)*
**Authoritative style score 20.00-66.67%**	108 (-0.08)	441 (0.17)	37 (-0.18)
**Authoritative style score 66.67-75.00%**	115 (-0.04)	460 (-0.70)	47 (1.29)
**Authoritative style score 75.00-81.67%**	97 (-0.40)	410 (0.57)	33 (-0.39)
**Authoritative style score 81.67-100%**	107 (0.51)	416 (-0.02)	32 (-0.77)
**Authoritarian style score 0-14.58%**	111 (0.02)	455 (0.72)	32 (-1.29)
**Authoritarian style score 14.58-22.92%**	116 (0.38)	464 (0.80)	29 (-2.00)
**Authoritarian style score 22.92-33.33%**	112 (0.30)	436 (-0.79)	43 (0.92)
**Authoritarian style score 33.33-83.33%**	88 (-0.73)	372 (-0.78)	45 (2.52)
**Permissive style score 0-18.75%**	121 (1.62)	441 (0.51)	20 (-3.44)

**Permissive style score 18.75-25.00%**	138 (1.72)	488 (-1.21)	40 (-0.58)
**Permissive style score 25.00-35.00%**	87 (-1.64)	420 (1.80)	32 (-0.57)
**Permissive style score 35.00-91.67%**	81 (-1.89)	378 (-1.03)	57 (4.80)
**Authoritative-training orientated style score 8.33-75.00%**	111 (-1.13)	488 (0.06)	51 (1.68)
**Authoritative-training orientated style score 75.00-87.50%**	141 (-0.54)	590 (0.06)	55 (0.74)
**Authoritative-training orientated style score 87.50-91.67%**	60 (0.79)	222 (-0.10)	15 (-1.07)
**Authoritative-training orientated style score 91.67-100%**	115 (1.16)	427 (-0.05)	28 (-1.74)
**Authoritative-intrinsic value orientated style score 4.17-50.00%**	123 (1.37)	447 (-0.57)	33 (-1.16)
**Authoritative-intrinsic value orientated style score 50.00-65.00%**	78 (-3.06)	432 (2.04)	42 (1.25)
**Authoritative-intrinsic value orientated style score 65.00-75.00%**	119 (0.92)	439 (-1.28)	43 (0.79)

**Authoritative-intrinsic value orientated style score 75.00-100%**	107 (0.70)	409 (-0.13)	31 (-0.87)
**Authoritarian-correction orientated style score 0-12.50%**	148 (0.85)	569 (0.06)	41 (-1.45)
**Authoritarian-correction orientated style score 12.50-21.88%**	99 (-0.63)	433 (1.38)	29 (-1.44)
**Authoritarian-correction orientated style score 21.88-31.25%**	88 (1.09)	318 (-0.73)	26 (-0.42)
**Authoritarian-correction orientated style score 31.25-84.38%**	92 (-1.30)	407 (-0.78)	53 (3.43)
