# Supplementary material for: Permissive parenting of the dog associates with dog overweight in a survey among 2,303 Dutch dog owners
Source: PLoS One. 2020 Aug 11;15(8):e0237429. doi: 10.1371/journal.pone.0237429 (PMC7418960; doi:10.1371/journal.pone.0237429)
Supplement: S3 Table — Dog overweight’s (including obesity) association with parenting styles was tested with logistic regression. The binary response variate of overweight discriminated Body Condition Scores (BCS) 6–9 (score 1) from BCS 0–5 (0). Regression models with single parenting styles only were extended with other possibly weight-explaining variables (exercise duration, dog age, dog sex/neutering status, owner educational level, owner age, owner gender), including two-way interactions with the styles. Full models with these seven explanatory variables and six interaction terms, were reduced by stepwise backwards elimination using Wald statistics, omitting the least significant term that was not marginal to another term at a threshold of P<0.05. For the pairwise comparisons for sex/neutering status we chose the lowest predicted mean of intact males as the reference. We present predicted mean fractions (±s.e.) for significant main effects/interactions (Wald test P<0.05) for the range of the 50% middle values (the two central quartiles; i.e. the range of common values) of each parenting style. Differences were significant for authoritarian (correction orientated), authoritative-training orientated and permissive parenting (P<0.05) in the single factor model and for authoritarian (correction orientated) and permissive parenting (interaction with dog sex/neutering status in the backwards elimination model (P<0.05)). (PDF) [file pone.0237429.s003.pdf]

S3 Table – Predicted mean fractions of overweight/obese dogs from logistic regressions with parenting styles and other explanatory variables

Dog overweight's (including obesity) association with parenting styles was tested with logistic regression. The binary response variate of overweight discriminated Body Condition Scores (BCS) 6-9 (score 1) from BCS 0-5 (0). Regression models with single parenting styles only were extended with other possibly weight-explaining variables (exercise duration, dog age, dog sex/neutering status, owner educational level, owner age, owner gender), including two-way interactions with the styles. Full models with these seven explanatory variables and six interaction terms, were reduced by stepwise backwards elimination using Wald statistics, omitting the least significant term that was not marginal to another term at a threshold of P<0.05. For the pairwise comparisons for sex/neutering status we chose the lowest predicted mean of intact males as the reference. We present predicted mean fractions (±s.e.) for significant main effects/interactions (Wald test P<0.05) for the range of the 50% middle values (the two central quartiles; i.e. the range of common values) of each parenting style. Differences were significant for authoritarian (correction orientated), authoritative-training orientated and permissive parenting (P<0.05) in the single factor model and for authoritarian (correction orientated) and permissive parenting (interaction with dog sex/neutering status in the backwards elimination model (P<0.05)).

**Parenting style** (range of common values in %)	**Single factor model** Predicted mean fractions for the range of common values (±s.e) (P-value)	**Backwards elimination model** P-value, predicted mean fractions for the range of common values (±s.e)
**Authoritative (67-82%)**	(P=0.704)	(P=0.739)
**Authoritarian (15-33%)**	0.05±0.01 to 0.07± 0.01 (P=0.006)	Parenting (P=0.006, from 0.04±0.01 to 0.05±0.01), exercise (P=0.003), dog sex/ neutering status (P=0.024), dog age (P<0.001), owner education (P=0.006), owner gender (P=0.048)
**Permissive (19-35%)**	0.05±0.01 to 0.07± 0.01 (P<0.001)	Parenting x sex/neutering status interaction (P=0.044, from 0.03±0.01 to 0.06±0.01 for intact ♀, 0.03±0.01 to 0.03±0.01 for intact ♂, 0.06±0.01 to 0.07±0.01 for neutered ♀, from 0.04±0.01 to 0.08±0.01 for neutered ♂[#]), exercise (P<0.001), dog age (P<0.001), owner education (P=0.012)
**Authoritative-training (75-92%)**	0.07±0.01 to 0.05± 0.01 (P<0.001)	Parenting (P=0.076, from 0.06±0.01 to 0.05±0.01), exercise (P=0.002), dog sex/neutering status (P=0.021), dog age (P<0.001), owner education (P=0.010)
**Authoritative-intrinsic value (50-75%)**	(P=0.362)	(P=0.368)
**Authoritarian-correction (13-31%)**	0.06±0.01 to 0.07± 0.01 (P=0.007)	Parenting (P=0.009, from 0.05±0.01 to 0.06±0.01), exercise (P=0.002), dog sex/neutering status (P=0.013), dog age (P<0.001), owner education (P=0.006)

[#]Pairwise comparisons with the reference of intact ♂: significant contrast between perm.♂intact and perm.♀intact (t-probability of P=0.038).
