# Supplementary material for: Permissive parenting of the dog associates with dog overweight in a survey among 2,303 Dutch dog owners
Source: PLoS One. 2020 Aug 11;15(8):e0237429. doi: 10.1371/journal.pone.0237429 (PMC7418960; doi:10.1371/journal.pone.0237429)
Supplement: S1 Appendix — For this survey-based research we tested the association between a dog owner’s parenting style directed at the dog and the dog’s body condition scores. Parenting style questionnaire items follow Van Herwijnen et al., 2018. Body condition scores were measured with pictures and descriptions of body condition scores-chart as propagated by the World Small Animal Veterinary Association (WSAVA) in their nutrition toolkit, and follow: https://www.wsava.org/WSAVA/media/Arpita-and-Emma-editorial/Body-Condition-Score-Dog.pdf. (PDF) [file pone.0237429.s004.pdf]

S4 Appendix - Questionnaire items

For this survey-based research we tested the association between a dog owner's parenting style directed at the dog and the dog's body condition scores. **Parenting style** questionnaire items follow Van Herwijnen et al., 2018*. **Body condition scores** were measured with pictures and descriptions of body condition scores-chart as propagated by the World Small Animal Veterinary Association (WSAVA) in their nutrition toolkit, and follow: https://www.wsava.org/WSAVA/media/Arpita-and-Emma-editorial/Body-Condition-Score-Dog.pdf.

**Reference: Van Herwijnen IR, van der Borg JA, Naguib M, Beerda B. The existence of parenting styles in the owner-dog relationship. PLoS ONE. 2018;13:e0193471.*

Parenting style questionnaire items follow Van Herwijnen et al., 2018:

I allow my dog to give input on decisions for instance with regard to the route we follow on walks.

I am responsive to my dog's feelings or needs

I can explode in anger towards my dog when he does something, he knows I don't want him to do.

I channel my dog's misbehaviour into a more acceptable activity.

I encourage my dog to 'be dog' even when it results in a dirty or wet dog.

I encourage my dog to show how it feels, it is allowed to growl for instance, when uncomfortable.

17 I find it difficult to discipline my dog.

18 I give comfort when my dog is upset.

19 I give into my dog when he causes a commotion about something or doesn’t do something, I want it to.

20 I give praise when my dog is good.

21 I grab my dog when he/she is being disobedient.

22 I have good times together with my dog.

23 I help my dog to understand the impact of its behaviour by offering him choices in situations.

24 I let my dog know how I feel about its good and bad behaviour.

25 I practice behaviour step by step with my dog, so I am sure he understands what I ask of him.

26 I practice certain behaviour with my dog before asking this behaviour in a more difficult situation.

27 I punish by giving my dog ‘time out’ and walking away if he misbehaves, even if he finds the situation he is in uncomforting.

28 I punish by taking away toys from my dog.

29 I raise my voice to make my dog improve.

30 I scold or criticize when my dog's behaviour doesn't meet my expectations.

31 I show respect for my dog' s needs by encouraging my dog to ‘be dog’.

32 I spoil my dog.

I take into account my dog's preferences in making plans.

I take my dog's desires into account before asking him to do something.

I think about why rules should be obeyed by my dog.

I threaten my dog with punishment more often than actually giving it.

I threaten with punishments towards my dog and do not actually do them.

I use a corrective slap when my dog misbehaves.

I use a poke of my finger, or short kick to snap my dog out of it when it misbehaves.

I use more or higher value reward (food or toy) when I believe my dog should really do something in a situation.

I use physical punishment (for instance a slap or a correction chain) as a way to improve my dog’s behaviour.

I use threats as punishment without feeling need for justification towards my dog.

I yell or shout when my dog misbehaves.

When I ask my dog to do something, he should do so, because I said so and I am its boss.

Body condition scores were measured with pictures and descriptions of body condition scores-chart as propagated by the World Small Animal Veterinary Association (WSAVA) in their nutrition toolkit, and follow: https://www.wsava.org/WSAVA/media/Arpita-and-Emma-editorial/Body-Condition-Score-Dog.pdf.

WSAVA
Global Nutrition Committee

Body Condition Score

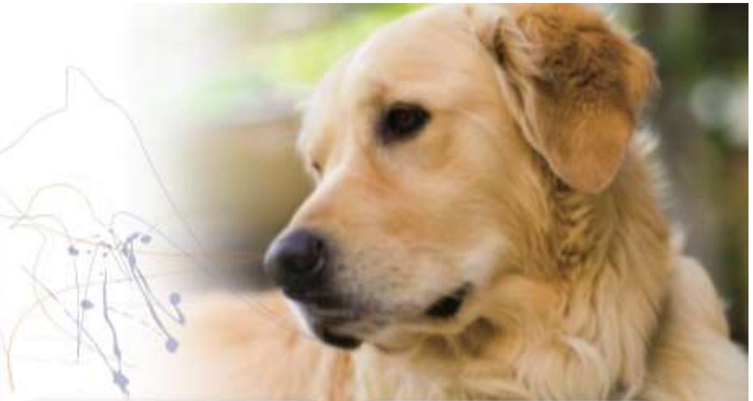

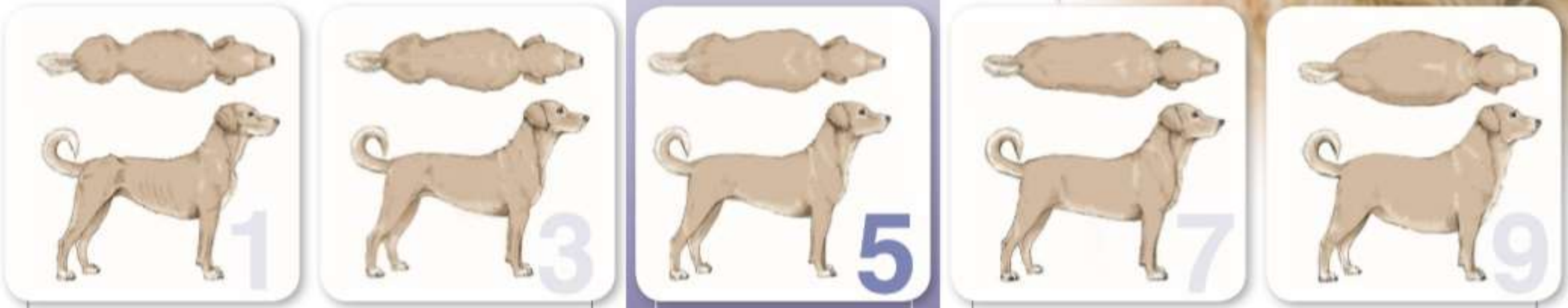

UNDER IDEAL

1. Ribs, lumbar vertebrae, pelvic bones and all bony prominences evident from a distance. No discernible body fat. Obvious loss of muscle mass.
2. Ribs, lumbar vertebrae and pelvic bones easily visible. No palpable fat. Some evidence of other bony prominences. Minimal loss of muscle mass.
3. Ribs easily palpated and may be visible with no palpable fat. Tops of lumbar vertebrae visible. Pelvic bones becoming prominent. Obvious waist and abdominal tuck.

IDEAL

4. Ribs easily palpable, with minimal fat covering. Waist easily noted, viewed from above. Abdominal tuck evident.
5. Ribs palpable without excess fat covering. Waist observed behind ribs when viewed from above. Abdomen tucked up when viewed from side.

OVER IDEAL

6. Ribs palpable with slight excess fat covering. Waist is discernible viewed from above but is not prominent. Abdominal tuck apparent.
7. Ribs palpable with difficulty; heavy fat cover. Noticeable fat deposits over lumbar area and base of tail. Waist absent or barely visible. Abdominal tuck may be present.
8. Ribs not palpable under very heavy fat cover, or palpable only with significant pressure. Heavy fat deposits over lumbar area and base of tail. Waist absent. No abdominal tuck. Obvious abdominal distention may be present.
9. Massive fat deposits over thorax, spine and base of tail. Waist and abdominal tuck absent. Fat deposits on neck and limbs. Obvious abdominal distention.

German A, et al. Comparison of a bioimpedance monitor with dual-energy x-ray absorptiometry for noninvasive estimation of percentage body fat in dogs. *AJVR* 2010;71:393-398.
Jeusette I, et al. Effect of breed on body composition and comparison between various methods to estimate body composition in dogs. *Res Vet Sci* 2010;88:227-232.
Kealy RD, et al. Effects of diet restriction on life span and age-related changes in dogs. *JAVMA* 2002;220:1315-1320.
Laflamme DP. Development and validation of a body condition score system for dogs. *Canine Pract* 1997;22:10-15.

©2013. All rights reserved.

wsava.org
