# Supplementary material for: Permissive parenting of the dog associates with dog overweight in a survey among 2,303 Dutch dog owners
Source: PLoS One. 2020 Aug 11;15(8):e0237429. doi: 10.1371/journal.pone.0237429 (PMC7418960; doi:10.1371/journal.pone.0237429)
Supplement: Attachment — Submitted filename: Response to Reviewers.docx [file pone.0237429.s006.docx]

Wageningen, February 16^th^ 2020

Dear reviewers,

We want to express our thanks for your constructive comments and feel we managed to process the comments effectively and/or address the issues in a logical and transparent way. We have revised the manuscript accordingly and please allow us to summarize how we responded to each point that was raised.

**Reviewer #1:**

1. Classification of body condition score (BCS)

We follow previous studies to classify the BCS into underweight, healthy-weight, overweight/obesity and list the references (line 52). In our study we do not determine whether high BCS associates with disease and a shorter lifespan. Merely we aim to study if and how BCS and dog-directed parenting associate (and we present information on our study approach in lines 145-158). The concern with regard to pre-categorising dogs into weight categories as to study health effects thus should not apply to our study. Furthermore, the implications of misclassifications are discussed in the Discussion (lines 284-298). We chose to analyse the data grouped in the often used underweight, healthy-weight, overweight/obesity classification as our participants dogs’ weights were not evenly spread over all BCS-categories, resulting in low counts in some categories and weakening the statistical power of the analysis. The reasons for merging BCS-categories is explained in the manuscript (lines 145-148).

2. Chi-square tests or linear regression models

It was suggested to use more sophisticated statistics to explain variation in BCS scores (i.e. by linear regression with multiple explanatory variables), but this approach does not fit the purpose of the study and would result in the testing of an arbitrary set of explanatory variables. The latter as the survey was not designed to record variables that likely explain variation in BCS, but functioned to establish the relation between BCS and dog-directed parenting. Adding complexity to the statistical analysis would in this case be in discord with the purpose of the study, introduce subjectivity and make the results less straightforward. Thus, we opted for Chi-square tests and argumentation for this choice is presented in the manuscript in lines 148-155.

3. Too much unrelated discussion

We removed lines 304-333 (previous submission) and generally shorted the Discussion section.

4. Questionnaire items

We added S3 Appendix, listing the parenting style questionnaire items.

5. Understandability of the Results section

We adapted the Results section, adding a Table and Figure, also responding to below ‘comment 9’.

6. Neglectful/uninvolved style

We added an explanation on not (being able to) study(ing) the uninvolved style (lines 127-129).

7. Sample size

Sample sizes were not estimated through power calculations before making the survey available online and we did not predetermine a survey period. However, 95% of the data was collected in the first three months after starting the survey advertisement and we added this information to the manuscript (lines 111-113).

8. Survey advertisement, aim mentioning

We now provide details on how participants were informed on the study aim (lines 96-101). Details on study advertisement can be found in lines 90-92.

9. Graph to visualize the data - BCS and/or weight of dogs

A graph on the dogs’ BCS was added to the Results section (Fig 1).

10. Paragraph for limitations

We address the limitations of 1) our study population’s characteristics in lines 277-288, of 2) owner-self report as method in lines 288-292, of 3) underestimation in lines 292-302.

Specific comments

The specific comments were processed by deleting or adapting the lines that were insufficiently clear. Also, we added the study duration to the Methods section (lines 110-111), made a note on the Dutch language (line 96), added a reference on residuals (lines 158-161) and we commented on the presumably long dog walks of our participants in the Discussion section (lines 282-284).

**Reviewer #2:**

Introduction:

We adapted the Introduction to create the suggested flow and removed the statements on One Health. Also, we shortened the section on dog-directed parenting styles and removed the details on the methods of the previous study. The aim was restated according to the feedback (lines 82-85).

Methods:

Details on replicates are now presented in lines 140-142.

Results:

A table replaces the previous text on the descriptive data of participants and their dogs (Table 1, starting line 174). Also, we moved the data on percentage and number of underweight, healthy-weight, overweight/obese dogs into this table. The suggestion for comparison with the European data was followed by adapting the Discussion in lines 284-288 and we thank the reviewer for pointing us to this reference.

Discussion:

We shorted the Discussion section, removing all information on weight loss. We address the limitations of 1) our study population’s characteristics in lines 277-288, of 2) owner-self report as method in lines 288-292, of 3) underestimation in lines 292-302.

 With these adaptations made to our manuscript, we expect to have addressed all points raised.
