# Supplementary material for: Permissive parenting of the dog associates with dog overweight in a survey among 2,303 Dutch dog owners
Source: PLoS One. 2020 Aug 11;15(8):e0237429. doi: 10.1371/journal.pone.0237429 (PMC7418960; doi:10.1371/journal.pone.0237429)
Supplement: Attachment — Submitted filename: commnets_20200317.docx [file pone.0237429.s007.docx]

Please see my comments as below.

Major points

1. The way that the authors responded to the reviewers’ comments was difficult for me to follow what you have changed during the last revision. It’s unclear whether the authors have addressed all the points raised by the reviewers.
2. The second comment that I made (Reviewer 1) was to run a multivariable linear model with the permissive style score as the outcome, which will answer the question that the authors intended to ask. Or you could run a multinomial logistic regression using the categories that you currently use in the Chi-square test. A big potential problem is that other variables such as sex, education level, walk time could even explain better your variation of the permissive style score than BCS, and you do have these pieces of information. Thus, I think this shouldn’t be ignored as what it is currently done in this manuscript.

Minor points

1. Lines 25-28: What is the hypothesis here?
2. Line 46: I am not sure about calling BCS measurement “subjective”. Yes, it’s rather subjective but it does have guidelines to be followed.
3. Lines 92-93: Is this an assumption or you provided guidelines and ensured that it was like this?
4. Lines 102-103: What did you do to make sure (a) only taken once and (b) not causing psychologically burdening?
5. I think Figure 1 has a better way to be presented. Also, this information was given in the table, and they were inconsistent.
