# Supplementary material for: Permissive parenting of the dog associates with dog overweight in a survey among 2,303 Dutch dog owners
Source: PLoS One. 2020 Aug 11;15(8):e0237429. doi: 10.1371/journal.pone.0237429 (PMC7418960; doi:10.1371/journal.pone.0237429)
Supplement: Attachment — Submitted filename: Response letter.docx [file pone.0237429.s008.docx]

Mrs. I. Anna S. Olsson, Ph.D.

Academic Editor PLOS ONE

1160 Battery Street

Koshland Building East, Suite 225

San Francisco, CA 94111

United States

Wageningen, July 26^th^ 2020

Dear Mrs. Olsson,

Thank you for the minor additional remarks.

*Line 101 Social desirability bias is still at play in anonymous online surveys, so please reword "so*

*without social pressure" to "hence minimizing social pressure"*

**Adjusted accordingly.**

*Line 112 Replace "it did not interfere" with "it was unlikely to interfere"*

**Adjusted accordingly.**

*Lines 342-343 This sentence is difficult to understand. Please consider rewording to "Weight*

*management of the dog could be one expression of dog-directed parenting, similarly to ...."*

**Adjusted accordingly.**

*Line 367 Please replace "which lacks in permissive parenting" with "which is low in permissive*

*parenting" (because demandingness is low rather than absent in permissive parenting, right?)*

**Adjusted accordingly.**

We also thank the reviewer for the final comment, recommending to set out a protocol up front and to perform a small pilot study first. We will take advantage of this advice when preparing future studies.

Sincerely,

Ir. Ineke R. van Herwijnen

Wageningen University

Behavioural Ecology Group, Department of Animal Sciences

P.O. Box 338

6700 AH Wageningen, The Netherlands
